# Effect of aspirin on short-term outcomes in hospitalized patients with COVID-19

**DOI:** 10.1177/1358863X211012754

**Published:** 2021-05-19

**Authors:** Aditya Sahai, Rohan Bhandari, Matthew Godwin, Thomas McIntyre, Mina K Chung, Jean-Pierre Iskandar, Hayaan Kamran, Essa Hariri, Anu Aggarwal, Robert Burton, Ankur Kalra, John R Bartholomew, Keith R McCrae, Ayman Elbadawi, James Bena, Lars G Svensson, Samir Kapadia, Scott J Cameron

**Affiliations:** 1 Section of Vascular Medicine, Department of Cardiovascular Medicine; Heart, Vascular & Thoracic Institute, Cleveland Clinic, Cleveland, OH, USA; 2 Lerner Research Institute, Cleveland Clinic, Cleveland, OH, USA; 3 Department of Cardiovascular Medicine; Heart, Vascular & Thoracic Institute, Cleveland Clinic, Cleveland, OH, USA; 4 Department of Internal Medicine, Cleveland Clinic, Cleveland, OH, USA; 5 Taussig Cancer Institute, Cleveland Clinic, Cleveland, OH, USA; 6 Division of Cardiovascular Medicine, University of Texas Medical Branch, Galveston, TX, USA; 7 Department of Quantitative Health Science, Cleveland Clinic, Cleveland, OH, USA

**Keywords:** ACE2, COVID-19, platelets, SARS-CoV-2, thrombosis, TMPRSS2

## Abstract

Coronavirus disease 2019 (COVID-19) caused by SARS-CoV-2 is an ongoing viral pandemic marked by increased risk of thrombotic events. However, the role of platelets in the elevated observed thrombotic risk in COVID-19 and utility of antiplatelet agents in attenuating thrombosis is unknown. We aimed to determine if the antiplatelet effect of aspirin may mitigate risk of myocardial infarction, cerebrovascular accident, and venous thromboembolism in COVID-19. We evaluated 22,072 symptomatic patients tested for COVID-19. Propensity-matched analyses were performed to determine if treatment with aspirin or nonsteroidal anti-inflammatory drugs (NSAIDs) affected thrombotic outcomes in COVID-19. Neither aspirin nor NSAIDs affected mortality in COVID-19. Thus, aspirin does not appear to prevent thrombosis and death in COVID-19. The mechanisms of thrombosis in COVID-19, therefore, appear distinct and the role of platelets as direct mediators of SARS-CoV-2-mediated thrombosis warrants further investigation.

## Introduction

COVID-19 is caused by the severe acute respiratory syndrome coronavirus-2 (SARS-CoV-2) and curiously displays a propensity for thrombosis in multiple vascular beds. COVID-19-related thrombosis may contribute to severe organ injury and death. The incidence of thrombotic events was as high as 31% in one cohort.^1^ Clinical and autopsy studies of patients with COVID-19 suggest an increased risk of microthrombi, venous thromboembolism (VTE), and ischemic stroke.^2,3^ Activated platelets are circulating mediators of thrombosis and, therefore, may serve as a logical therapeutic target in COVID-19. Several registered clinical trials will prospectively evaluate patient outcomes following low-dose aspirin in the context of SARS-CoV-2 infection, but high-quality observational data in the interim are lacking.

SARS-CoV-2 utilizes a spike glycoprotein to bind to the host transmembrane angiotensin-converting enzyme 2 (ACE2), then is cleaved by the serine protease TMPRSS2 to coordinate entry into the host cell.^4,5^ Therefore, co-expression of ACE2 and TMPRSS2 may be important for host cell entry and infectivity of SARS-CoV-2. Importantly, human tissue distribution of ACE2 and TMPRSS2 mirrors organ system involvement in COVID-19 and includes the lungs,^6–11^ vascular endothelium,^9–12^ heart,^11,13,14^ kidneys,^8,10,13^ liver,^8,10^ digestive tract,^8,10,11,15^ nasal epithelium,^7,10,11^ and central nervous system.^10,14^

Single-stranded RNA (ssRNA) viruses, including influenza, are engulfed by platelets and may contribute to immuno-thrombosis indirectly through developing neutrophil extracellular traps (NETs) by engaging the platelet toll-like receptor 7 (TLR7).^16^ SARS-CoV-2, another ssRNA virus, utilizes platelets to modulate immunologic responses including in the development of NETs, which emerged as a particularly important prothrombotic response in patients with COVID-19.^17^ Furthermore, elevation of soluble P-selectin and sCD40L in blood from patients with COVID-19 compared to controls provides indirect evidence of platelet activation in COVID-19 coagulopathy.^18^ SARS-CoV-2 is a ssRNA virus, and therefore may directly augment platelet activation causing myocardial infarction (MI), stroke, and VTE.

A recent report demonstrated that patients with COVID-19 have a divergent platelet transcriptome compared with healthy individuals, and aspirin suppresses COVID-19 platelet activation in vitro.^19^ The platelet surface receptor for SARS-CoV-2 was not clarified in this study, while a similar investigation by another group identified mRNA for SARS-CoV-2 in human platelets.^20^ Thus, in the absence of prospective clinical trial data, we sought to evaluate the potential benefit of mitigating thrombotic responses *in vivo* with use of aspirin or other nonsteroidal anti-inflammatory drug (NSAID) antiplatelet therapies by propensity matching patients using real-world data.

## Materials and methods

### Study design

Institutional review board approval was obtained to evaluate de-identified patient data, thus informed patient consent was not required. Clinical data from ambulatory and hospitalized Cleveland Clinic patients treated in Northeast Ohio and South Florida were appraised from 22,072 symptomatic patients evaluated for COVID-19 with the goal of determining if current aspirin use protects patients from death and/or the secondary composite outcome of MI, thrombotic stroke, and/or VTE. Stringent quality assurance checks for data integrity and abstraction occurred continuously throughout the study as indicated in the online supplemental material. Positive testing for a SARS-CoV-2 amplicon by nasopharyngeal reverse transcriptase-polymerase chain reaction (RT-PCR) was used to determine infection status. The electronic medical record (EMR) and hospital medication administration record (MAR) were used to confirm new or ongoing administration of 81 mg aspirin or other NSAIDs for both outpatients and inpatients. The timeframe for medications was defined as being started prior to testing for SARS-CoV-2 and ended after testing or, if not discontinued, started within 90 days prior to testing.

### Statistical analysis

Categorical factors are summarized using frequencies and percentages, while continuous factors are described using median and ranges. Initial descriptive analyses were performed. Comparisons were made between those with known death status and those with missing death information to identify if any differences exist in these cohorts. Then amongst those with known death status, differences in COVID positive and COVID negative patients were assessed. Finally, after stratifying by COVID status, comparisons of those with and without aspirin use were performed. For all tables, continuous measures were compared using nonparametric Wilcoxon rank sum tests, while categorical factors were compared using Pearson chi-squared tests or Fisher’s exact tests, for rare events.

Given the differences across many covariates, propensity score matching was performed to account for differences between those with and without aspirin use. This approach used two steps. First, multiple imputation was performed on all demographic and covariate measures within COVID status stratified datasets, using fully conditional specification methods. The multiple imputation process for the clinical registry accounted for 10–20% of missing data to better match the groups, following the procedures described previously in a similar investigation.^21^ Ten imputed datasets were created. Then, propensity score models were fit for each dataset, with aspirin use as the response and all other measures as predictors. The predicted probability of aspirin use from each model was calculated, and these probabilities were averaged across models for each patient. Greedy matching was then performed using a caliper of 0.2 SDs of the logit to create matched datasets for both COVID positive and negative patients. A small number of aspirin users could not be matched well and were excluded from the matched analysis. Comparisons of outcomes were performed using mixed effect logistic regression models to account for the matching process. Overlap weighting propensity score analyses were also performed with the same conclusions drawn from the data, as shown.^22^ This analysis was repeated using NSAID groups. For significant effects, E-values that represent the magnitude of the association between an unobserved covariate and both the medication group and outcome necessary to make the result non-significant were also calculated.^23^ These were post hoc analyses in which the primary outcome was in-hospital mortality. The secondary outcomes were stroke, MI, and VTE individually, then as a composite thrombotic secondary endpoint. To account for a NSAID class effect rather than an effect caused by aspirin, the same propensity matching was conducted to study the effect of NSAIDs as some prior reports appeared to suggest a signal for harm in patients with COVID-19.^24^ The following medications were considered NSAIDs in this analysis: aspirin, diflunisal, dexibuprofen, naproxen, fenoprofen, ketoprofen, dexketoprofen, indomethacin, tolmetin, sulindac, etodolac, ketorolac, diclofenac, piroxicam, meloxicam, tenoxicam, droxicam, lornoxicam, mefenamic acid, meclofenamic acid, flufenamic acid, tolfenamic acid, celecoxib, and ibuprofen. Analyses were performed using SAS software (version 9.4; SAS Institute Inc., Cary, NC, USA). A significance level of 0.01 was used for all tests.

## Results

**Figure 1.**
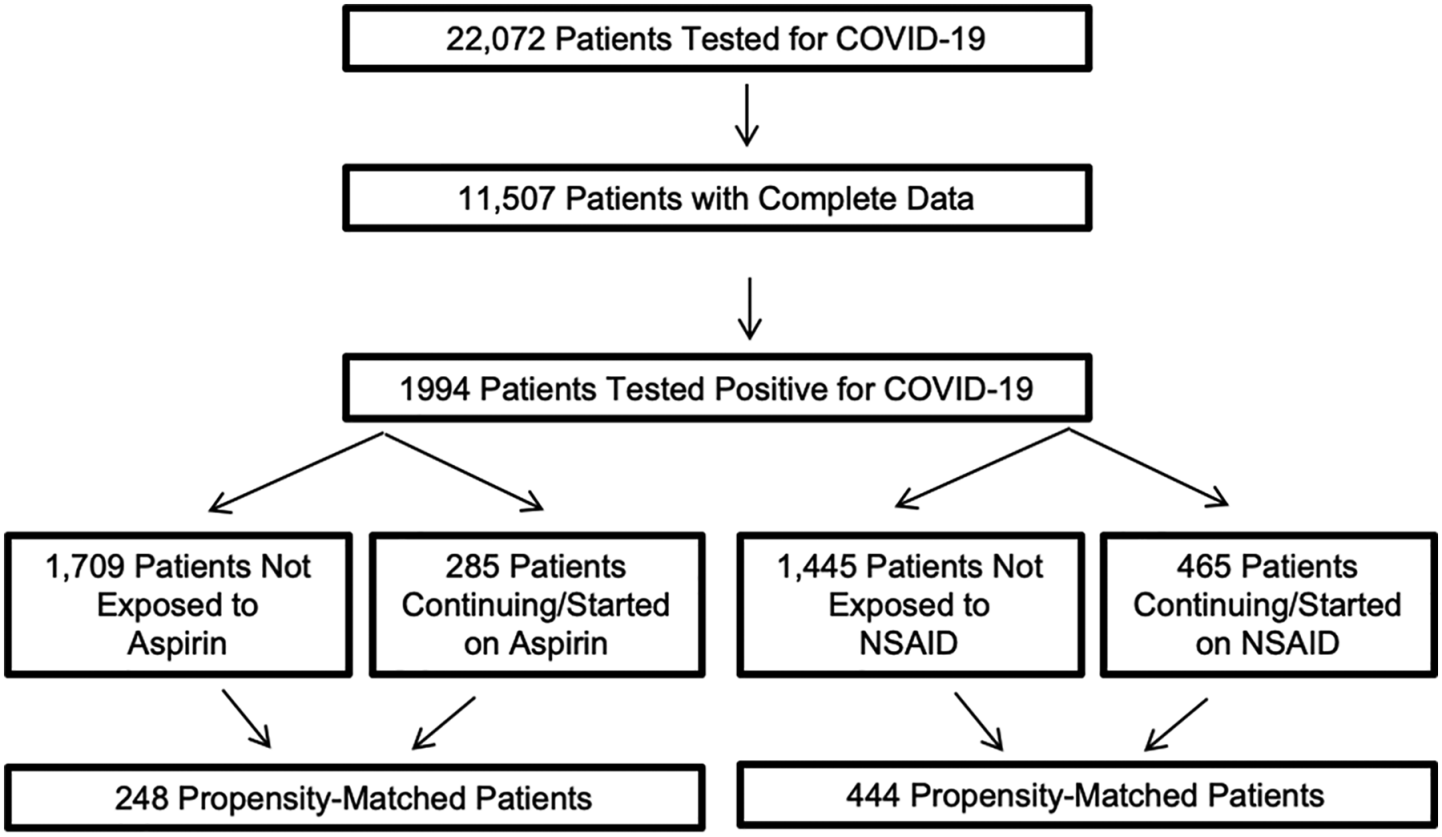
Patients testing positive for COVID-19 taking aspirin or NSAIDs. Patients testing positive for a SARS-CoV-2 amplicon at two Cleveland Clinic hospitals were evaluated. Patients initiated with aspirin or NSAID therapy or continuing aspirin or NSAID if admitted to the hospital were included in this study. Clinical variables in each group were then re-evaluated following careful propensity matching. COVID-19, coronavirus disease 2019; NSAID, nonsteroidal anti-inflammatory drug; SARS-CoV-2, severe acute respiratory syndrome coronavirus-2.

A total of 22,072 patients tested for COVID-19 at two Cleveland Clinic hospitals between March 13, 2020 and May 13, 2020 were evaluated. Within this cohort, 11,507 patients had complete clinical data and 1994 tested positive for the SARS-CoV-2 amplicon by RT-PCR testing. Amongst these 1994 patients, 1709 were not exposed and 285 patients were exposed to aspirin. In an attempt to differentiate an antiplatelet drug effect with aspirin from a more general NSAID class effect, 1445 patients not exposed and 465 patients exposed to NSAID therapy were propensity-matched (Figure 1).

**Figure 2.**
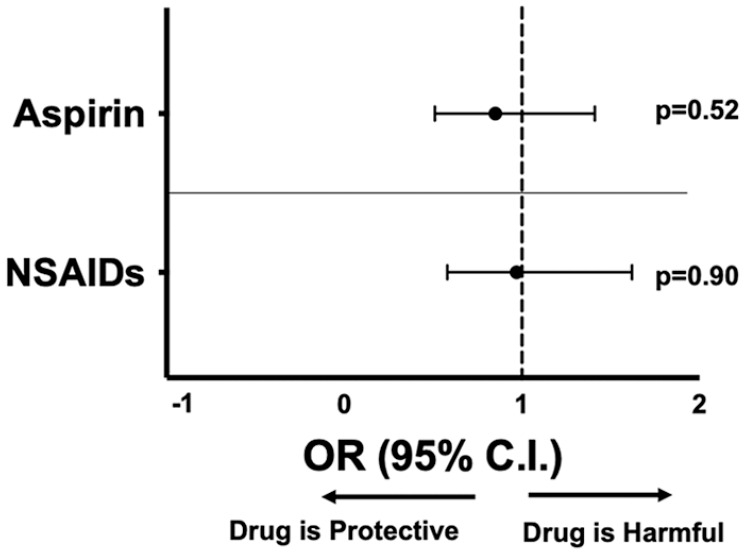
Mortality for propensity-matched patients. Propensity-matched data for patients testing positive for COVD-19 and outcomes taking either 81 mg aspirin (*n* = 248 in each group) or NSAIDs (*n* = 444 in each group) at the time of diagnosis. Forest plot representation of data as OR with 95% CI for the primary endpoint of death. NSAID, nonsteroidal anti-inflammatory drug; OR, odds ratio.

Table 1 shows the unadjusted characteristics of each comparative cohort for aspirin. The 248 propensity-matched patients either treated with aspirin or not, demonstrated no significant group differences in demographics or clinical covariates (online supplemental Figure S1). Aspirin therapy did not alter mortality (13.3% vs 15.3%, *p* = 0.53). The 444 propensity-matched patients either exposed or not to NSAIDs demonstrated no significant group differences in demographics or clinical covariates (online supplemental Figure S2). NSAID therapy did not alter mortality (7.0% vs 7.2%). In propensity-matched patients treated with aspirin, the incidence of MI (2.0% vs 0.81%) and VTE (4.0% vs 1.6%) were not different, but aspirin therapy was associated with a higher incidence of thrombotic stroke (3.6% vs 0.40%). Using the composite thrombotic endpoint of MI, VTE, and thrombotic stroke, aspirin was associated with more thrombotic events (9.3% aspirin vs 2.8% no aspirin; *p* = 0.005) (Table 2). In propensity-matched patients treated with NSAIDs, the incidence of MI (0.68% vs 0.23%), VTE (2.0% vs 0.90%), and thrombotic stroke (1.1% vs 0.45%) was not significantly different individually or as a combined endpoint (Table 3). Overall, there was no change in mortality for patients with COVID-19 treated with aspirin (OR 0.85, 95% CI: 0.51–1.41; *p* = 0.52) or NSAIDs (OR 0.97, 95% CI: 0.58–1.62; *p* = 0.90) (Figure 2). To assess if those previously on aspirin therapy had a predilection for thrombotic events and resultingly may have an increased risk of recurrent thrombosis during SARS-CoV-2 infection compared to those without history of thrombosis, we evaluated relevant thrombotic history in those patients hospitalized with SARS-CoV-2 infection taking aspirin. Of the five patients taking aspirin with in-hospital MI after COVID-19 diagnosis, one had a prior Type I MI and one had a prior thrombotic stroke. Of the nine patients taking aspirin with in-hospital stroke after COVID-19 diagnosis, one had a prior thrombotic stroke and one had a prior MI. Of the 10 patents taking aspirin with in-hospital VTE after COVID-19 diagnosis, none had a prior MI and none had a prior stroke. Of the three patients taking NSAIDs with in-hospital MI after COVID-19 diagnosis, one had a prior MI and one had a prior thrombotic stroke. Of the five patients taking NSAIDs with in-hospital stroke after COVID-19 diagnosis, one had a prior MI and none had a prior stroke. Of the nine patients taking NSAIDs with in-hospital VTE after COVID-19 diagnosis, none had a prior MI and none had a prior stroke.

**Table 1.** Baseline patient population for aspirin use: clinical and demographic data for patients testing positive for SARS-CoV-2 not taking aspirin or with established aspirin therapy or initiated on low-dose aspirin (81 mg) at the time of diagnosis.

Factor	No aspirin		Aspirin use		*p*-value
	*N*	*n* (%)	*N*	*n* (%)	
**Medications**					
Clopidogrel	1709	9 (0.53)	285	27 (9.5)	**<0.001** ^a^
Ticagrelor	1709	1 (0.06)	285	6 (2.1)	**<0.001** ^b^
Prasugrel	1709	0 (0.00)	285	0 (0.00)	
Cangrelor	1709	0 (0.00)	285	0 (0.00)	
Cilostazol	1709	0 (0.00)	285	0 (0.00)	
Pentoxifylline	1709	0 (0.00)	285	1 (0.35)	0.14^b^
All antiplatelet agents	1709	10 (0.59)	285	285 (100.0)	**<0.001** ^a^
Multiple therapy	1709	0 (0.00)	285	34 (11.9)	**<0.001** ^b^
Therapeutic anticoagulation	1709	94 (5.5)	285	56 (19.6)	**<0.001** ^a^
Prophylactic anticoagulation	1709	355 (20.8)	285	215 (75.4)	**<0.001** ^a^
NSAIDs	1650	294 (17.8)	260	171 (65.8)	**<0.001** ^a^
**Covariates**					
Age	1709	50.6 ± 17.5	285	70.0 ± 13.6	**<0.001** ^c^
Platelets	689	217.4 ± 79.3	253	208.7 ± 85.3	0.14^d^
Sex	1651		285		**<0.001** ^a^
Male		804 (48.7)		172 (60.4)	
Female		847 (51.3)		113 (39.6)	
Race	1564		280		**<0.001** ^a^
White		948 (60.6)		144 (51.4)	
Black		506 (32.4)		124 (44.3)	
Other		110 (7.0)		12 (4.3)	
Ethnicity	1480		277		**<0.001** ^a^
Hispanic		204 (13.8)		7 (2.5)	
Non-Hispanic		1276 (86.2)		270 (97.5)	
Smoking	1417		268		**<0.001** ^a^
No		924 (65.2)		123 (45.9)	
Former		362 (25.5)		124 (46.3)	
Current		131 (9.2)		21 (7.8)	
Respiratory support	1709	191 (11.2)	285	117 (41.1)	**<0.001** ^a^
Pressors	1709	81 (4.7)	285	47 (16.5)	**<0.001** ^a^
Hemodynamic instability	1709	85 (5.0)	285	48 (16.8)	**<0.001** ^a^
COPD	1399	82 (5.9)	274	53 (19.3)	**<0.001** ^a^
Asthma	1410	243 (17.2)	273	66 (24.2)	**0.007** ^a^
Diabetes	1424	318 (22.3)	278	147 (52.9)	**<0.001** ^a^
Hypertension	1447	659 (45.5)	281	244 (86.8)	**<0.001** ^a^
Coronary artery disease	1405	116 (8.3)	275	100 (36.4)	**<0.001** ^a^
Heart failure	1404	108 (7.7)	274	78 (28.5)	**<0.001** ^a^
Cancer	1447	184 (12.7)	280	63 (22.5)	**<0.001** ^a^
Immunosuppressive treatment	1456	144 (9.9)	277	36 (13.0)	0.12^a^
Transplant history	1403	11 (0.78)	271	8 (3.0)	**0.006** ^b^
Multiple sclerosis	1403	14 (1.00)	272	6 (2.2)	0.12^b^
Connective tissue disease	1401	127 (9.1)	273	44 (16.1)	**<0.001** ^a^
Inflammatory bowel disease	1397	65 (4.7)	271	14 (5.2)	0.72^a^
Immunosuppressive disease	1398	159 (11.4)	272	71 (26.1)	**<0.001** ^a^

Statistically significant *p* values are indicated in bold.

a Pearson’s chi-squared test; ^b^Fisher’s exact test; ^c^Satterthwaite *t*-test; ^d^*t*-test.

COPD, chronic obstructive pulmonary disease; NSAID, nonsteroidal anti-inflammatory drug; SARS-CoV-2, severe acute respiratory syndrome coronavirus-2.

**Table 2.** Propensity-matched outcomes for aspirin use: clinical and demographic data for patients testing positive for SARS-CoV-2 not taking aspirin or with established aspirin therapy or initiated on low-dose aspirin (81 mg) at the time of diagnosis.

Factor	No aspirin (*n* = 248)	Aspirin use (*n* = 248)	*p*-value
	*n* (%)	*n* (%)	
Thrombotic stroke	1 (0.40)	9 (3.6)	0.036
MI	2 (0.81)	5 (2.0)	0.27
VTE	4 (1.6)	10 (4.0)	0.12
Secondary composite (death, thrombotic stroke, MI, VTE)	7 (2.8)	23 (9.3)	**0.005**

Statistically significant *p* values are indicated in bold.

MI, myocardial infarction; SARS-CoV-2, severe acute respiratory syndrome coronavirus-2; VTE, venous thromboembolism.

**Table 3.** Propensity-matched outcomes for NSAID use: clinical and demographic data for patients testing positive for SARS-CoV-2 not taking aspirin or with established NSAID therapy or initiated on NSAID therapy at the time of diagnosis.

Factor	No (*n* = 444)	Yes (*n* = 444)	*p*-value
	*n* (%)	*n* (%)	
Thrombotic stroke	2 (0.45)	5 (1.1)	0.27
MI	1 (0.23)	3 (0.68)	0.34
VTE	4 (0.90)	9 (2.0)	0.17
Secondary composite (death, thrombotic stroke, MI, VTE)	7 (1.6)	17 (3.8)	0.046

MI, myocardial infarction; NSAID, nonsteroidal anti-inflammatory drug; SARS-CoV-2, severe acute respiratory syndrome coronavirus-2; VTE, venous thromboembolism.

## Discussion

In this study, we found that treatment with low-dose aspirin failed to provide protection from death or thrombotic outcomes in patients with COVID-19. This observation may be related to the dose administered, an insensitivity to aspirin’s mechanism of platelet inhibition in COVID-19, or an altered platelet phenotype. Evidence of a deranged and altered platelet phenotype in COVID-19 was demonstrated by Manne et al. Further, Cameron et al. have previously demonstrated a divergent platelet phenotype in patients with chronic arterial disease and diabetes leading to diminished host responses to aspirin and clopidogrel in diseased platelets.^25^ Similarly, Hu et al. demonstrated in platelets from patients with diabetes, surface P2Y_12_ receptors are arranged in a different conformation and are impressively resistant to inhibition by clopidogrel.^26^ These observations demonstrate antiplatelet agents’ responses can be altered in the host based upon environmental conditions and it may be the case that aspirin is less effective in patients with COVID-19 for similar reasons.

Recent investigations revealed platelet reactivity is enhanced in patients with COVID-19 and appears to be suppressed by high-dose aspirin in vitro.^20,27,28^ In the absence of randomized controlled data for aspirin use in patients with COVID-19, we conducted a propensity-matched analysis of patients taking aspirin. We conclude that aspirin has no overall mortality benefit in this retrospective observational study of patients with COVID-19, and eagerly await the data from appropriately powered randomized, controlled studies using antiplatelet agents – especially the Protective Effect of Aspirin on COVID-19 (PEAC) trial and the Randomised Evaluation of COVID-19 Therapy (RECOVERY) trial.^29^ Platelet reactivity data *in vitro* are often extrapolated to suggest a risk for harm, but it is important to acknowledge that the behavior of antiplatelet medications *in vivo* can be markedly different from *ex vivo* studies. Our goal was to clarify this concern by using real-life data with both mortality and thrombotic end points.

Elbadawi *et al.* reported the absolute neutrophil count and not D-dimer, a traditional biomarker associated with thrombosis, is an independent predictor of thrombotic events in patients with COVID-19.^30^ The mortality benefit of dexamethasone, an immunosuppressant and anti-inflammatory medication, in hospitalized patients with COVID-19^31^ and recent reports of immunothrombosis^17,32–35^ and microvascular occlusion^18,36–38^ by multiple independent groups, suggest platelets may be indirect mediators of thrombosis and perhaps not the best direct targets for pharmacological intervention. Contemporaneous with submission of this manuscript, a smaller, nonpropensity-matched study has shown aspirin treatment decreased mortality that was driven by reduced ICU level care and mechanical ventilatory needs but not thrombosis in patients with COVID-19. This report suggests a protective effect of aspirin that is distinct from altering end-organ thrombosis,^39^ and possibly from immune-mediated acute respiratory distress syndrome (ARDS) as previously demonstrated.^40,41^ By evaluating another anti-inflammatory mechanism using patients treated with NSAIDs in parallel with aspirin in the same hospital and locations in the US, we similarly show no effect on mortality, with all statistical models accounting for any contribution of prophylactic and therapeutic heparin use in hospitalized patients and subsequent outcomes.

The signal for increased composite thrombotic events in patients with COVID-19 treated with aspirin was surprising and driven mostly by stroke, likely suggesting an increased baseline risk in these patients and hence the reason the patients may have been on aspirin therapy. Recent observational studies show mixed results for COVID-19-related stroke risk with one small study suggesting an increased risk in younger patients,^42^ one large study showing an overall low risk,^43^ and one very large study paradoxically showing that COVID-19 infection is associated with a decreased risk of thrombotic cerebrovascular stroke.^44^ A mechanistic explanation for these observations is entirely speculative, though aspirin does reduce production of interleukin-6 (IL-6), a cytokine with demonstrated neuroprotective effects.^45,46^

Zaid *et al.* identified SARS-CoV-2 mRNA in human platelets, implying a mechanism of entry must exist, and then a report by Zhang et al. identified ACE2 on human platelets.^20,47^ These data are at odds with Manne et al. who failed to detect ACE2 protein in platelets by immunoblotting using only white blood cells (WBCs) as a positive control. Notably, Manne *et al.* employed a CD45 depletion step on isolated platelets to eliminate the possibility of WBC contamination prior to immunoblotting. CD45 is also present on platelets, and we previously demonstrated this step decreases the platelet yield available for immunoblotting.^48^ Lastly, Nassa et al. have very elegantly shown that the platelet transcriptome and proteome are dynamic and often mRNA to protein concordance is not observed but, rather, dependent on external platelet cues.^49^

The observational and retrospective nature of this study from just two hospitals has clear intrinsic limitations, and the small patient sample to allow for propensity matching greatly limits generalizability of our findings. These data are exploratory and hypothesis-generating, and we make no claims regarding the potential effectiveness or limitations of aspirin in protecting patients with COVID-19 from thrombotic events including MI and stroke. In addition, thrombotic stroke and MI were relatively rare events in our population of patients with COVID-19, with a very small number of those patients with a prior history of MI and stroke. Therefore, aspirin therapy may simply be a coincidental signal that a patient already has a higher risk of thrombosis and so requires this medication.

## Conclusions

Our real-world clinical data suggest regular intake of low-dose aspirin does not protect against adverse thrombotic events or death in patients with COVID-19. Platelets are fastidious components of the circulatory system with a wide range of critical functions, including contributing to immunoinflammatory host responses. Thus, targeting platelet thrombotic function may alter its roles in other domains. The nuanced mechanisms of thrombosis in COVID-19 may be unique and deserve further investigation. The use of traditional antiplatelet agents may not protect against thrombotic events or mortality in COVID-19, but, in fact, cause harm. The awareness of this potential harm and role of randomized controlled drug trials in assessing the suitability of antiplatelet agents in COVID-19 is critical.

## Supplemental Material

sj-docx-1-vmj-10.1177_1358863X211012754 – Supplemental material for Effect of aspirin on short-term outcomes in hospitalized patients with COVID-19 Click here for additional data file. Supplemental material, sj-docx-1-vmj-10.1177_1358863X211012754 for Effect of aspirin on short-term outcomes in hospitalized patients with COVID-19 by Aditya Sahai, Rohan Bhandari, Matthew Godwin, Thomas McIntyre, Mina K Chung, Jean-Pierre Iskandar, Hayaan Kamran, Essa Hariri, Anu Aggarwal, Robert Burton, Ankur Kalra, John R Bartholomew, Keith R McCrae, Ayman Elbadawi, James Bena, Lars G Svensson, Samir Kapadia and Scott J Cameron in Vascular Medicine
