# Supplementary material for: Effect of aspirin on short-term outcomes in hospitalized patients with COVID-19
Source: Vasc Med. 2021 May 19;26(6):626–32. doi: 10.1177/1358863X211012754 (PMC8137864; doi:10.1177/1358863X211012754)
Supplement: sj-docx-1-vmj-10.1177_1358863X211012754 – Supplemental material for Effect of aspirin on short-term outcomes in hospitalized patients with COVID-19 [file sj-docx-1-vmj-10.1177_1358863X211012754.docx]

**Supplemental File**

**Methods**

The COVID-19 Registry Quality Assurance (QA) model at the Cleveland Clinic was designed to uphold standards of data entry, security, and validation. Data is collected and stored using a secure database with complete audit trail and access limited to study team members. To ensure appropriate data validation, data is analyzed using gold standard statistical software. Data science experts ensure proper statistical methodology is being utilized, assist with cross-project coordination, statistically validate data before release, and review COVID projects prior to publication for completion. While the quality standards of data entry, security, and validation have been maintained throughout the life of the registry, the QA goals and implementation have evolved from a process of reviewing manually entered data to the utilization of optimized automated algorithms to pull accurate information into the database, eliminating the need for human data entry/review. The COVID-19 Research Registry QA team began with a “Reviewer” group of 40 and a “QA” group of 18 medical students, research coordinators, and research nurses. The reviewers were responsible for manually abstracting and entering a subset of variables from patient records that could not initially be automatically extracted from the electronic health record (EHR). Reviewers were also asked to verify high-priority variables that had been automatically pulled into the database from the EHR. The QA group was responsible for verifying select data variables – whether automatically or manually abstracted. Training was done remotely as new members joined the research registry team. Resources provided included a pre-recorded discussion of COVID-19 science and rationale for the research registry and a user guide offering detailed instructions as well as production expectations. Team members who had experience with a particular patient population were directed to focus on that group (i.e. pediatric patients, patients with multiple sclerosis, patients who are admitted to the hospital/ICU). Data entry revisions on manual and auto-populated variables were tracked and reviewed, along with comments logged in the database or sent via email by reviewer and QA groups. Revisions and comments were reviewed by members of the registry administrative team, including the PI and Data Scientists, for identification of any trends requiring re-education/training, solicitation of subject-matter experts or improvement of the natural language processing used to help better define an auto-populated variable that consistently required revision, or a practical change to the database (i.e. adding descriptive labels to clarify collection/interpretation instructions or highlight key information such as COVID-positive status). Areas of QA focus have been flexible and assigned based on current global priority, including specific patient populations and specific forms in the Registry database. Initially, the QA group performed standard practice of 30% QA on all records in the database comprised of over 300 variables per record. Over a period of seven months, the QA group reviewed roughly 50% of the 15,000+ then-available patient records in the Registry. At that point, focus shifted to validation of the algorithms used to automate data entry. The QA group verified 10-15% of patient records containing these select automated variables and utilized this information to inform/optimize the algorithms. The accuracy of the updated algorithms was then assessed to determine whether another set of records needed to be reviewed or if mathematically accurate results had been achieved programmatically such that human review was no longer necessary. To continue testing our algorithms, the QA group began reviewing roughly 200 patient records each month. Initial analyses identified human to automation agreement at or below 30% for some variables. Through an iterative process lasting three months, agreement rates improved to upwards of 90% for all variables. Our QA group conducts a spot-check of 100 records in their entirety once every two months to ensure our human to automation agreement remains at a threshold of 85% or above.

**Table S1. Propensity-matched Patient Population for Aspirin Use**

| **Factor** | **No Aspirin (n=248)** | **Aspirin (n=248)** | **OR (95% CI)** | **p-value** |
| --- | --- | --- | --- | --- |
| ***Medications*** |  |  |  |  |
| Clopidogrel | 5 (2.0) | 20 (8.1) |  |  |
| Ticagrelor | 0 (0.00) | 5 (2.0) |  |  |
| Prasugrel | 0 (0.00) | 0 (0.00) |  |  |
| Cangrelor | 0 (0.00) | 0 (0.00) |  |  |
| Cilostazol | 0 (0.00) | 0 (0.00) |  |  |
| Pentoxifylline | 0 (0.00) | 1 (0.40) |  |  |
| All Anti-platelet agents | 5 (2.0) | 248 (100.0) |  |  |
| Multiple Anti-platelet agents | 0 (0.00) | 26 (10.5) |  |  |
| Therapeutic Anticoagulation | 47 (19.0) | 43 (17.3) | 0.90 (0.57, 1.4) | 0.64 |
| Prophylactic Anticoagulation | 179 (72.2) | 178 (71.8) | 0.98 (0.66, 1.5) | 0.92 |
| NSAIDs | 38 (16.3) | 151 (66.8) | 10.3 (6.6, 16.1) | ***<0.001*** |
| ***Covariates*** |  |  |  |  |
| Age | 69.5 ± 14.1 | 68.5 ± 13.6 | 0.99 (0.98, 1.01) | 0.42 |
| Sex |  |  |  |  |
| Male | 147 (59.5) | 140 (56.5) | reference |  |
| Female | 100 (40.5) | 108 (43.5) | 1.1 (0.79, 1.6) | 0.49 |
| Race |  |  |  |  |
| White | 137 (57.8) | 122 (50.2) | reference |  |
| Black | 92 (38.8) | 109 (44.9) | 1.3 (0.92, 1.9) | 0.13 |
| Other | 8 (3.4) | 12 (4.9) | 1.7 (0.67, 4.3) | 0.27 |
| Ethnicity |  |  |  |  |
| Hispanic | 6 (2.5) | 7 (2.9) | reference |  |
| Non-Hispanic | 230 (97.5) | 235 (97.1) | 0.88 (0.29, 2.6) | 0.81 |
| Smoking |  |  |  |  |
| No | 113 (49.3) | 112 (47.9) | reference |  |
| Former | 101 (44.1) | 101 (43.2) | 1.01 (0.69, 1.5) | 0.96 |
| Current | 15 (6.6) | 21 (9.0) | 1.4 (0.69, 2.9) | 0.34 |
| Respiratory Support | 98 (39.5) | 95 (38.3) | 0.95 (0.66, 1.4) | 0.78 |
| Pressors | 43 (17.3) | 41 (16.5) | 0.94 (0.59, 1.5) | 0.81 |
| Hemodynamic Instability | 44 (17.7) | 42 (16.9) | 0.95 (0.59, 1.5) | 0.81 |
| COPD | 30 (13.6) | 45 (18.7) | 1.5 (0.88, 2.4) | 0.14 |
| Asthma | 40 (18.3) | 60 (24.9) | 1.5 (0.94, 2.3) | 0.091 |
| Diabetes | 112 (50.0) | 123 (50.4) | 1.02 (0.71, 1.5) | 0.93 |
| Hypertension | 204 (85.0) | 208 (84.9) | 0.99 (0.60, 1.6) | 0.97 |
| Coronary Artery Disease | 58 (26.1) | 70 (28.9) | 1.2 (0.76, 1.7) | 0.50 |
| Heart Failure | 51 (22.9) | 62 (25.8) | 1.2 (0.77, 1.8) | 0.46 |
| Cancer | 58 (25.0) | 55 (22.4) | 0.87 (0.57, 1.3) | 0.51 |
| Immunosuppressive Therapy | 31 (13.4) | 32 (13.2) | 0.98 (0.58, 1.7) | 0.95 |
| History of Transplant | 4 (1.8) | 8 (3.3) | 1.9 (0.56, 6.3) | 0.31 |
| Multiple Sclerosis | 3 (1.4) | 6 (2.5) | 1.8 (0.45, 7.4) | 0.39 |
| Connective Tissue Disease | 32 (14.5) | 40 (16.7) | 1.2 (0.71, 1.9) | 0.53 |
| IBD | 9 (4.1) | 11 (4.6) | 1.1 (0.46, 2.8) | 0.80 |
| Immunosuppressive Agents | 50 (23.1) | 65 (27.2) | 1.2 (0.81, 1.9) | 0.32 |

Propensity-matched data for n=248 patients in each group testing positive for SARS-CoV-2 not taking aspirin or with established aspirin therapy or initiated on low dose aspirin (81 mg) at the time of diagnosis. The composite endpoint is: MI, thrombotic stroke, VTE.

**Table S2. Propensity-matched Patient population for NSAID Use**

| **Factor** | **No NSAIDs (n=444)** | **NSAIDs (n=444)** | **OR (95% CI)** | **p-value** |  |
| --- | --- | --- | --- | --- | --- |
| ***Medications*** |  |  |  |  |  |
| Clopidogrel | 8 (1.8) | 18 (4.1) |  |  |  |
| Ticagrelor | 0 (0.00) | 4 (0.90) |  |  |  |
| Prasugrel | 0 (0.00) | 0 (0.00) |  |  |  |
| Cangrelor | 0 (0.00) | 0 (0.00) |  |  |  |
| Cilostazol | 0 (0.00) | 0 (0.00) |  |  |  |
| Pentoxifylline | 0 (0.00) | 1 (0.23) |  |  |  |
| All Anti-platelet agents | 58 (13.1) | 156 (35.1) | 3.6 (2.6, 5.1) | ***<0.001*** |  |
| Multiple anti-platelet agents | 3 (0.68) | 21 (4.7) | 7.3 (2.2, 24.6) | ***0.001*** |  |
| Therapeutic Anticoagulation | 51 (11.5) | 43 (9.7) | 0.83 (0.54, 1.3) | 0.38 |  |
| Prophylactic Anticoagulation | 200 (45.0) | 182 (41.0) | 0.85 (0.65, 1.1) | 0.22 |  |
| ***Covariates*** |  |  |  |  |  |
| Age | 58.2 ± 18.1 | 58.1 ± 17.0 | 1.00 (0.99, 1.01) | 0.93 |  |
| Platelets | 204.7 ± 82.7 | 212.8 ± 77.6 | 1.00 (1.00, 1.00) | 0.24 |  |
| Gender |  |  |  |  |  |
| Male | 216 (49.8) | 227 (51.5) | reference |  |  |
| Female | 218 (50.2) | 214 (48.5) | 0.93 (0.72, 1.2) | 0.61 |  |
| Race |  |  |  |  |  |
| White | 214 (51.2) | 233 (54.2) | reference |  |  |
| Black | 179 (42.8) | 171 (39.8) | 0.88 (0.66, 1.2) | 0.36 |  |
| Other | 25 (6.0) | 26 (6.0) | 0.96 (0.54, 1.7) | 0.88 |  |
| Ethnicity |  |  |  |  |  |
| Hispanic | 25 (6.2) | 31 (7.2) | reference |  |  |
| Non-Hispanic | 376 (93.8) | 402 (92.8) | 0.86 (0.50, 1.5) | 0.60 |  |
| Smoking |  |  |  |  |  |
| No | 219 (55.3) | 243 (56.1) | reference |  |  |
| Former | 136 (34.3) | 149 (34.4) | 0.99 (0.73, 1.3) | 0.93 |  |
| Current | 41 (10.4) | 41 (9.5) | 0.90 (0.56, 1.4) | 0.66 |  |
| Respiratory Support | 92 (20.7) | 79 (17.8) | 0.83 (0.59, 1.2) | 0.27 |  |
| Pressors | 34 (7.7) | 26 (5.9) | 0.75 (0.44, 1.3) | 0.29 |  |
| Hemodynamic Instability | 37 (8.3) | 28 (6.3) | 0.74 (0.44, 1.2) | 0.25 |  |
| COPD | 47 (12.1) | 46 (10.9) | 0.89 (0.58, 1.4) | 0.61 |  |
| Asthma | 80 (20.4) | 94 (22.3) | 1.1 (0.80, 1.6) | 0.51 |  |
| Diabetes | 142 (35.9) | 153 (35.7) | 0.99 (0.74, 1.3) | 0.93 |  |
| Hypertension | 269 (66.1) | 271 (62.7) | 0.86 (0.65, 1.1) | 0.31 |  |
| Coronary Artery Disease | 75 (19.2) | 82 (19.2) | 1.00 (0.71, 1.4) | 0.99 |  |
| Heart Failure | 62 (15.8) | 62 (14.6) | 0.91 (0.62, 1.3) | 0.63 |  |
| Cancer | 65 (16.1) | 70 (16.4) | 1.02 (0.70, 1.5) | 0.93 |  |
| Immunosuppressive Treatment | 53 (13.1) | 53 (12.4) | 0.94 (0.62, 1.4) | 0.76 |  |
| Transplant History | 5 (1.3) | 9 (2.1) | 1.7 (0.55, 5.0) | 0.36 |  |
| Multiple Sclerosis | 4 (1.04) | 9 (2.1) | 2.1 (0.63, 6.7) | 0.23 |  |
| Connective Tissue Disease | 43 (11.1) | 58 (13.6) | 1.3 (0.83, 1.9) | 0.28 |  |
| Inflammatory Bowel Disease | 11 (2.9) | 20 (4.7) | 1.7 (0.80, 3.6) | 0.17 |  |
| Immunosuppressive Disease | 63 (16.3) | 75 (17.7) | 1.1 (0.76, 1.6) | 0.61 |  |
| Propensity-matched data for n=444 patients testing positive for SARS-CoV-2 and outcomes for NSAID use at the time of diagnosis. The composite endpoint is: MI, thrombotic stroke, VTE.  Statistics presented as Mean ± SD, N (column %). Odds Ratios (OR), Confidence Intervals (CI) and p-values correspond to univariate logistic regression models. | | | | | |
